# Oral administration of rumen-protected choline to ewes during the periconceptional period skews the sex ratio of lambs

**DOI:** 10.1530/RAF-25-0139

**Published:** 2026-03-02

**Authors:** Masroor Sagheer, Quinn A Hoorn, Daniel Carballo, Mariangela B C Maldonado, Jade A Paxton, Brittany N Diehl, Azmeera Thirupathi, Thainá Minela, Joseph W McFadden, Ky G Pohler, Peter J Hansen

**Affiliations:** ^1^ Department of Animal Sciences, Institute of Food and Agricultural Sciences, D H Barron Reproductive and Perinatal Biology Research Program, and Genetics Institute, University of Florida, Gainesville, Florida, USA; ^2^ EARTH University, Guácimo, Costa Rica; ^3^ Department of Large Animal Clinical Sciences, University of Florida, Gainesville, USA; ^4^ Department of Animal Science, Cornell University, Ithaca, New York, USA; ^5^ Department of Animal Science, Texas A&M University, College Station, Texas, USA

**Keywords:** choline, programming, sex ratio, sheep

## Abstract

**Graphical Abstract:**

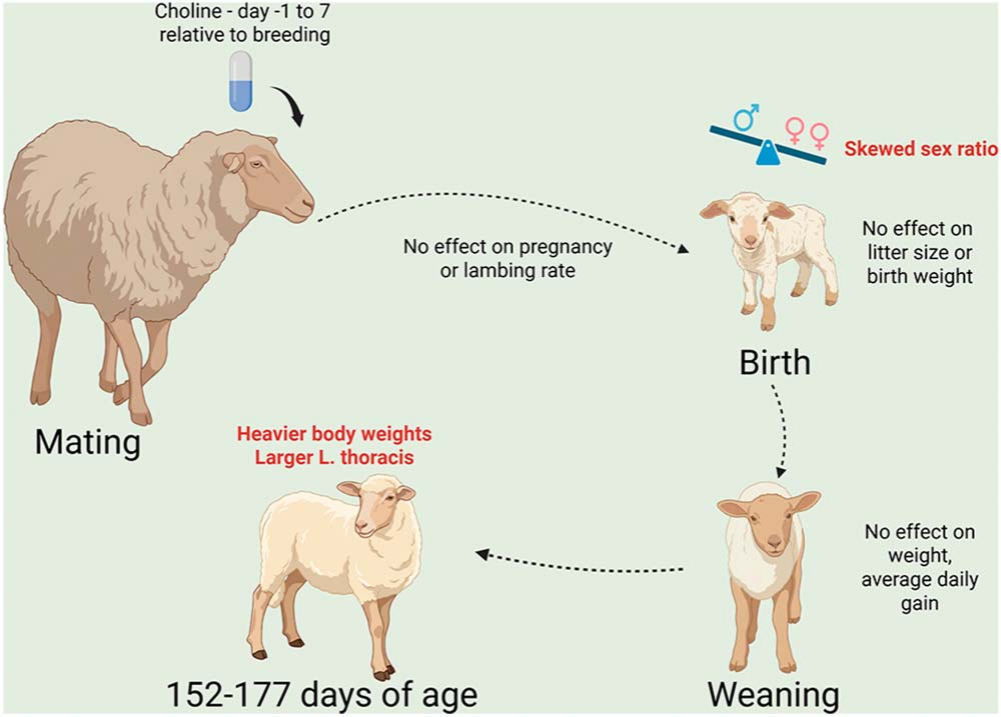

**Abstract:**

Feeding rumen-protected choline (RPC) to beef cows during the periconceptional period has been reported to alter embryonic development to reduce weaning weight of the resultant calves. Here, it was tested whether oral administration of RPC to ewes during the periconceptional period programs postnatal development of lambs. In a preliminary experiment, oral administration of 5 g RPC (representing 3 g choline chloride) tended to increase plasma concentrations of the choline metabolite trimethylamine *N*-oxide, but the treatment did not affect concentrations of any other metabolite, including choline itself. In a subsequent experiment, 183 ewes were treated with oral administration of RPC or a control capsule daily from day −1 to day 7 after anticipated breeding. Rumen-protected choline did not affect pregnancy or lambing rate, plasma concentrations of placenta-associated glycoproteins, or litter size but skewed the sex ratio toward females. Treatment with RPC did not affect lamb weight at birth or through weaning (∼83 days of age) or testis weight at castration (∼50 days of age). For a subset of lambs examined at an average age of 169 days, RPC increased body weight and cross-sectional area and depth of the longissimus thoracis muscle. In summary, maternal supplementation of RPC during the periconceptional period shifted the sex ratio toward females and increased post-weaning body weight and size of the longissimus thoracis muscle area of the resultant lambs. These data provide further evidence for environmental modification of sex ratio in mammals and illustrate the impact that specific nutrients early in development can have on postnatal phenotype.

**Lay summary:**

Modification of the mother’s environment early in pregnancy has been reported to alter characteristics of the resultant offspring. Here, it was shown that feeding a form of the nutrient choline that escapes destruction in a ewe’s stomach influenced the sex ratio of the lambs (increasing the fraction that were female) and increased the cross-sectional area of a major muscle in the lab. Results provide further evidence for environmental modification of sex ratio in mammals and illustrate the impact specific nutrients early in development can have on the characteristics of the offspring after birth.

## Introduction

The periconceptional period refers to the time before and after conception, encompassing ovulation, fertilization, and early embryo development. During this window, the early embryo undergoes key processes, such as embryonic genome activation, epigenetic reprogramming, mitochondrial activation, and lineage commitment (Hansen & Tríbulo 2019, Zhu *et al.* 2021). Changes in the maternal environment during this period can affect development of the preimplantation embryo to have lasting effects on offspring development and health (Hansen *et al.* 2016, Hansen & Siqueira 2018, Velazquez *et al.* 2023).

One group of molecules that can influence early embryonic development is the class of metabolites involved in one-carbon metabolism. This biochemical network supplies methyl groups through the methionine and folate cycles to support cellular processes, such as nucleotide and protein synthesis, redox balance, mitochondrial function, and epigenetic regulation (Xu & Sinclair 2015, Ducker & Rabinowitz 2017). Key metabolites include methionine, choline, betaine, folate, and B vitamins. Changes in the availability of molecules involved in one-carbon metabolism during the periconceptional period can alter embryo development. Supplementing oocyte maturation medium with a cocktail of molecules involved in one-carbon metabolism reduced DNA fragmentation, increased DNMT3A protein expression, and improved blastocyst yield in cattle (Golestanfar *et al.* 2022, 2025). Addition of the folate antagonist methotrexate to culture medium blocked embryonic development in cattle and ewes (Kwong *et al.* 2010), while disruption of methionine metabolism with ethionine prevented blastocyst development in mice (Kudo *et al.* 2015) and cattle (Ikeda *et al.* 2012). Altering concentrations of methionine in culture medium of bovine embryos resulted in changes in the characteristics of the resultant blastocyst, including changes in lipid content and trophectoderm numbers (Sagheer *et al.* 2025*a*). Alterations in one-carbon metabolism during early development can also change the developmental program of the embryo to alter postnatal phenotype. Supplementation of rumen-protected methionine for 14 days around the time of ovulation resulted in increased stature in the preweaning period in calves from supplemented dams (Heredia *et al.* 2025).

Choline is a nutrient involved in multiple metabolic pathways, including those integral to one-carbon metabolism. It serves as a precursor for phosphatidylcholine (a key component of cell membranes), acetylcholine (a neurotransmitter involved in muscle contraction and neural function), and the methyl donor S-adenosyl methionine (Jiang *et al.* 2014). Addition of choline chloride to embryo culture medium increased DNA methylation in bovine blastocysts (Estrada-Cortés *et al.* 2020) and altered postnatal characteristics of the resultant calves, including heavier weaning weight, paired testes weight, and carcass weight, as well as altered DNA methylation profiles in muscle and whole blood (Estrada-Cortés *et al.* 2021, Haimon *et al.* 2024*a*,*b*). These findings suggest that maternal choline concentrations during early embryonic development could affect the health and productivity of the offspring.

Choline metabolism in ruminants is complex due to its ruminal degradation. Free choline is rapidly converted to trimethylamine by rumen microbes, which is subsequently converted into trimethylamine *N*-oxide (TMAO) in the liver by flavin-containing monooxygenase 3, reducing the amount of choline available for metabolism (Zeisel *et al.* 1989). Rumen-protected choline (RPC) products have been developed that can partially bypass ruminal degradation, although they do not always elevate plasma choline concentrations (De Veth *et al.* 2016, Zenobi *et al.* 2018, France *et al.* 2022, Lima *et al.* 2024). In cattle, providing supplemental choline during the periconceptional period through feeding of RPC from one day before until seven days after timed artificial insemination did result in changes in the phenotype of the resultant calves but in an opposite manner to that caused by providing choline to cultured bovine embryos (Sagheer *et al.* 2025*b*). In particular, feeding RPC to females reduced body weight, hip height, and weight-to-hip ratio of their calves at four months of age and weaning. These outcomes contrast with the effects observed with direct choline chloride supplementation in embryo culture medium cited earlier, suggesting that feeding choline exerts different effects on the embryo than those caused by culture in choline-containing medium. These additional effects could include alterations in concentrations of specific choline metabolites or changes in rumen function.

Effects of RPC supplementation during the periconceptional period in ewes remain unexplored. Therefore, the objective of this study was to evaluate whether feeding RPC to ewes during the periconceptional period alters the postnatal phenotype of lambs. It was hypothesized that feeding RPC from day −1 to +7 relative to anticipated natural breeding would program the embryo in a manner that decreases postnatal growth of lambs. An initial experiment was performed to assess changes in plasma concentrations of choline and its metabolites following RPC supplementation. Subsequently, the effects of RPC supplementation during the periconceptional period on the postnatal development of lambs were assessed.

## Materials and methods

### Animal care

All procedures involving animals were approved by the Animal Care and Use Committee of the University of Florida (protocol 201910942) and were performed following the relevant guidelines and regulations.

### Circulating concentrations of choline and its metabolites after oral administration of rumen-protected choline

The experiment was designed to quantify the plasma concentrations of free choline and its metabolites after oral administration of a single bolus of RPC (ReaShure, Balchem Inc., USA; 60% choline chloride (w/w)). A total of eight adult ewes housed in a single pasture were blocked by body weight and randomly divided into RPC (*n* = 4) and control (*n* = 4) groups. Ewes in the RPC groups were individually administered a single oral bolus of 5 g RPC (3 g choline chloride), while ewes in the control group received an empty bolus at the same time. The 5 g RPC was packaged in a single gelatin capsule (Torpac, Fairfield, NJ, USA) before oral administration. The ewes were restrained by placing a hand under the face to move the head slightly upward, while the balling gun (Torpac) with the gelatin capsule was gently inserted in the gap between the molar and incisor teeth. The capsule was dispensed on the back of the tongue, and each animal was monitored to confirm that it swallowed the capsule. Blood samples (5 mL) were collected by jugular venipuncture before (0 h) and 2, 4, 6, 8, and 10 h after bolus administration into EDTA-containing tubes and immediately centrifuged at 3,000 ***g*** for 15 min to obtain the blood plasma fraction. Plasma samples were stored at −80°C until further analysis. Concentrations of choline, betaine, methionine, dimethylglycine, trimethylamine, and TMAO in plasma were determined using an Agilent Technologies 1260 Infinity-II Liquid Chromatography system with a thermostated autosampler coupled with 6,460 tandem mass spectrometry and the data processed with MassHunter, version 10.0 (Holm *et al.* 2003).

### Programming of postnatal development by oral administration of rumen-protected choline in the periconceptional period

The experiment was designed to test whether oral administration of RPC (ReaShure; 60% choline chloride (w/w)) during the periconceptional period can affect pregnancy outcomes and alter the postnatal development of the resultant lambs. The experiment was performed in three replicates. A total of 183 ewes of various breeds (Katahdin, Florida Native, Dorper, Awassi, and crossbreeds) were available for the experiment (*n* = 53, 93, and 37 for replicates 1, 2, and 3). Replicates 1 (matings in August 2022) and 2 (matings in August 2023) were conducted at one location in Florida with Katahdin, Florida Native, and crossbred ewes. Replicate 3 was conducted at a distinct Florida location (matings in May 2024) with Dorper and Awassi ewes. For each replicate, ewes within each breed were blocked by body weight and assigned randomly within the block to the RPC (*n* = 95) or control group (*n* = 88). Ewes were synchronized with a 7-day CIDR protocol for timed natural breeding. Ewes received an intravaginal CIDR device (0.3 g progesterone, Eazi-Breed CIDR Sheep Insert, Zoetis Inc., USA) on day −9 of the protocol. On day −2, the CIDR was removed and 250 μg prostaglandin F2α (Estrumate®, Merck, USA) was injected, i.m. On the same day, rams were mixed with ewes. The day of anticipated breeding was day 0. Ewes were assigned to rams (*n* = 7 for replicates 1 and 2; *n* = 3 for replicate 3) as per the standard breeding program of the farms. Rams were separated from ewes after 56 days of introduction.

The ewes were individually administered an oral bolus of 5 g RPC or an empty gelatin capsule (control) each day from day −1 until day 7 after anticipated breeding. This represents a period corresponding to final follicular growth, ovulation, fertilization, and embryonic development until the blastocyst stage of development. Pregnancy diagnosis was performed by transabdominal ultrasonography at days 30 and 84 (replicates 1 and 2 only) and days 30 and 92 (replicate 3) after anticipated breeding. Only the ewes found pregnant at day 30 were kept in the experiment.

For replicates 1 and 2, blood samples (5 mL) were collected by jugular venipuncture at days 30 and 84 relative to breeding into EDTA-containing tubes to quantify the plasma concentration of pregnancy-associated glycoproteins (PAGs) in pregnant ewes. Samples were collected on days 30 and 84 relative to breeding (replicate 1) or day 30 (replicate 2). The total number of samples was 85 for day 30 and 36 for day 84. Blood samples were immediately centrifuged after collection at 3,000 ***g*** for 15 min to obtain the plasma fraction. Plasma samples were stored at −80°C until further analysis. All plasma samples were subjected to an in-house-validated sandwich enzyme-linked immunosorbent assay for quantification of PAGs (Pohler *et al.* 2016). The intra- and inter-assay coefficients of variation were 4.0 and 4.7%, respectively.

For replicates 1 and 2, ewes had continuous access to bahiagrass pasture and free-choice access to a mineral supplement. During the breeding season and post-partum period (through weaning), the ewes received a commercial concentrate ration of 16% crude protein, fed at a rate of 2% of body weight per day, as well as free-choice access to Coastal Bermuda grass hay. For replicate 3, ewes had continuous access to bahiagrass pasture and Tifton 44 hay, a sheep mineral mix free choice, and a grain supplement of 17.8% crude protein fed at 1% of body weight per day. During the breeding season, the grain supplement was fed at 2% of body weight per day and the hay was replaced with Tifton 44 hay mixed with molasses.

Pregnant ewes were kept under similar managemental conditions until lambing. Data on lamb sex, litter size, and birth weight were collected at lambing. Lambs were weighed twice a month until weaning (average age: 83 days). At an average age of 50 days, male lambs for replicates 1 and 2 were castrated and paired testes weight was recorded after removing all external connective tissue. At an average age of 169 days (range: 152–177 days), carcass ultrasound was performed on the right side of each lamb (only in replicate 2). Images of muscle were obtained by a certified technician using a real-time ultrasound machine (IBEX EVO III, USA), equipped with a linear transducer (3–6 MHz), 30 cm depth and standoff. Wool and hair were clipped from the scan site to an approximate length of 3.2 mm. Warm mineral oil was used as a conductive medium. Settings for the ultrasound device were 15 dB for gain, 10 dB for near, 25 dB for far, 10 cm for depth, and 100 for field of view. The measurements taken were the cross-sectional area and depth of the *longissimus thoracis* muscle (between the 12th and 13th ribs) and the subcutaneous fat thickness at the same location.

### Statistical analysis

Data were analyzed using SAS, version 9.4 (SAS Institute, Cary, NC, USA). For experiment 1, plasma choline metabolite concentrations were analyzed using analysis of variance with the MIXED procedure. The model included fixed effects of treatment, time, and their interaction, with ewe as a random effect and time specified as a repeated measure.

For experiment 2, response variables were first analyzed using full statistical models and then reanalyzed to improve parsimony by removing effects in the model that explained small amounts of variance. Concentrations of PAGs were measured by analysis of variance using the PROC GLM procedure of SAS. The full model for PAG concentrations at day 30 included effects of treatment, replicate, litter size, and all two-way interactions, while the full model for PAG concentrations at day 84 included treatment, litter size, and the interaction. Pregnancy outcomes (pregnancy and lambing rates), sex ratio and postnatal mortality were analyzed using logistic regression with the GLIMMIX procedure of SAS, employing a logit link function and binary distribution and with Kenward–Roger adjustments for degrees of freedom. Model fit was evaluated by calculating the Pearson chi-square statistic divided by degrees of freedom. The full statistical model for pregnancy outcomes included effects of treatment, dam breed, the interaction between treatment and breed, replicate, and the interaction between treatment and replicate. Values for model fit ranged from 1.02 to 1.03. The full statistical model for sex ratio included effects of treatment, dam breed, the interaction between treatment and breed, the interaction between replicate and treatment, litter size (1 vs > 1), the interaction between litter size and treatment, and the random effect of litter nested within treatment and breed. The value for model fit was 0.93. The statistical model for postnatal mortality was similar, except litter size, which was removed to allow convergence of the model. The model fit was 0.46. Litter size was analyzed by the GLIMMIX procedure using a log function and negative binomial distribution. Data on birth weight, postnatal weights through 93 days of age, and average daily gain between birth and weaning were analyzed by analysis of variance using the MIXED procedure. The full statistical model included treatment, dam breed, replicate, sex, litter size (1 vs > 1), all two-way interactions, age as a covariate, and the random effect of litter nested within treatment and dam breed. Carcass measurements via ultrasound were analyzed with a similar model, except that replicate and its interactions were not in the model. Testis weight was analyzed using a model that included treatment, dam breed, litter size (1 vs > 1), all two-way interactions, and the random effect of litter nested within treatment and breed.

## Results

### Circulating concentration of choline and its metabolites after oral administration of rumen-protected choline

**Figure 1:**
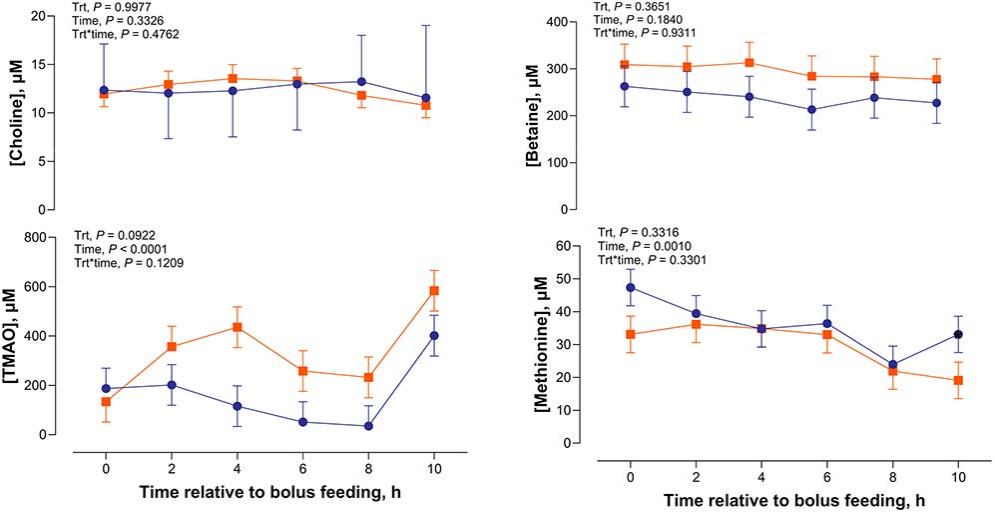
Effect of a single oral bolus of rumen-protected choline on plasma concentrations of choline and selected metabolites. The blue lines represent control, and the orange lines represent RPC. Data are least-squares mean ± SEM.

Changes in blood plasma concentrations of choline and associated metabolites after oral administration of RPC bolus are shown in Fig. 1. There was no significant effect of treatment (*P* = 0.998), time (*P* = 0.335), or the treatment by time interaction (*P* = 0.476) on plasma concentrations of choline, indicating that plasma choline concentrations remained stable across time and were unaffected by RPC administration. In contrast, plasma TMAO concentrations were affected by time (*P* < 0.0001), with concentrations fluctuating across the 10 h period following bolus feeding. There was a tendency (*P* = 0.092) for a treatment effect, with greater concentrations in the RPC group relative to the control group. There was no treatment by time interaction (*P* = 0.121). Plasma betaine concentrations were not influenced by treatment (*P* = 0.365), time (*P* = 0.184), or the interaction (*P* = 0.931). Plasma methionine concentrations declined over time in both groups (*P* =0.001) but were not affected by treatment (*P* = 0.332) or the interaction of treatment and time (*P* = 0.330).

### Characteristics of lambs at birth as affected by oral administration of rumen-protected choline to females from day −1 to day 7 after anticipated breeding

There was no effect of feeding of RPC on the proportion of ewes becoming pregnant on day 30 or 84/92 post-breeding or on the proportion of ewes lambing (Table 1). Similarly, treatment did not affect pregnancy loss between days 30 and 84/92 of gestation, day 84/92 and lambing, or between day 30 and lambing (Table 1).

**Table 1:** Effect of oral administration of rumen-protected choline (RPC) to ewes from day −1 to day 7 after anticipated breeding on pregnancy outcomes and plasma concentrations of pregnancy-associated glycoproteins (PAGs).

Item	Treatment		Adj. OR (RPC/choline)	95% CI	*P* ^†^
	Control	RPC			
Proportion of ewes pregnant, *n*/*n* (%)					
Day 30	57/88 (64.8)	61/95 (64.2)	1.17	0.59–2.33	0.656
Days 84–92*	54/88 (61.4)	57/95 (60.0)	1.13	0.58–2.22	0.713
Proportion of ewes lambed, *n*/*n* (%)	53/88 (60.2)	54/95 (56.8)	1.05	0.54–2.04	0.888
Pregnancy loss, *n*/*n* (%)					
Days 30–84/92	3/57 (5.3)	4/61 (6.6)	1.26	0.27–6.00	0.767
Day 84/92 – lambing	1/54 (1.9)	3/57 (5.26)	2.94	0.29–29.98	0.358
Day 30 – lambing	4/57 (7.0)	7/61 (11.5)	1.72	0.47–6.30	0.411

* Pregnancy diagnosed on day 84 after anticipated mating in replicates 1 and 2 and day 92 after anticipated mating in replicate 3.

^†^ Statistical comparison of treatment.

Adj. OR, adjusted odds ratio; CI, confidence interval.

Feeding RPC did not affect concentrations of PAGs in plasma at day 30 (0.55 ± 0.10 ng/mL for control vs 0.60 ± 0.11 ng/mL for RPC; *P* = 0.733) or 84 (1.78 ± 0.26 ng/mL for control vs 1.32 ± 0.28 ng/mL for RPC; *P* = 0.242). Note that there was an effect of litter size on PAG concentrations at both day 30 (*P* = 0.024) and day 84 of gestation (*P* = 0.023). In both cases, concentrations were lower for ewes with singleton pregnancies than for ewes with more than one fetus. Least-squares means were 0.41 ± 0.10 vs 0.74 ± 0.11 ng/mL for singleton vs ≥ 2 fetuses at day 30 and 1.10 ± 0.26 vs 2.01 ± 0.28 ng/mL at day 84.

As shown in Table 2, litter size (*P* = 0.892) was not different between the control and RPC groups. The percent of litters that were singletons, twins, and triplets were 58.5, 37.7, and 3.8% for control and 53.7, 42.6, and 3.7% for RPC. Treatment with RPC skewed the sex ratio (*P* = 0.020), with the adjusted odds ratio of a female lamb being 2.48 for RPC as compared to control. The percent of lambs that were female was 44% for control (34/77) and 62% for RPC (*n* = 50/81). The difference in sex ratio was apparent for both singleton and twin pregnancies (interaction, *P* = 0.275). The percent of lambs that were female for singleton pregnancies was 42% (13/31) for control and 72% (21/29) for RPC. The percent of lambs that were female for pregnancies with ≥2 lambs was 46% (21/46) for control and 56% (29/52) for RPC.

**Table 2:** Effect of oral administration of rumen-protected choline (RPC) from day −1 to day 7 after anticipated breeding on selected characteristics of the resultant lambs.

Item	Treatment		Adj. OR (RPC/choline)	95% CI	*P*-value
	Control	RPC			
Total lambs, *n*	77	81	-	-	-
Mean litter size, n*	1.6 ± 0.1	1.7 ± 0.1	-	-	0.829
Sex ratio, *n*/*n* (% female)	34/77 (44)	50/81 (62)	2.48	1.16–5.31	0.020
Postnatal mortality, *n*/*n* (%)^†^	4/77 (5.2)	4/81 (4.9)	1.01	0.22–4.64	0.982

* The percent of litters that were singletons, twins, and triplets were 58.5, 37.7, and 3.8% for control and 53.7, 42.6, and 3.7% for RPC.

^†^ Mortality was 0/31 singleton lambs for control and 1/29 singleton lambs for RPC; 3/40 twin lambs for control and 3/46 twin lambs for RPC; and 1/6 triplet lambs for control and 0/6 triplet lambs for RPC.

### Postnatal development of lambs through weaning as affected by oral administration of rumen-protected choline to females from day −1 to day 7 after anticipated breeding

**Figure 2:**
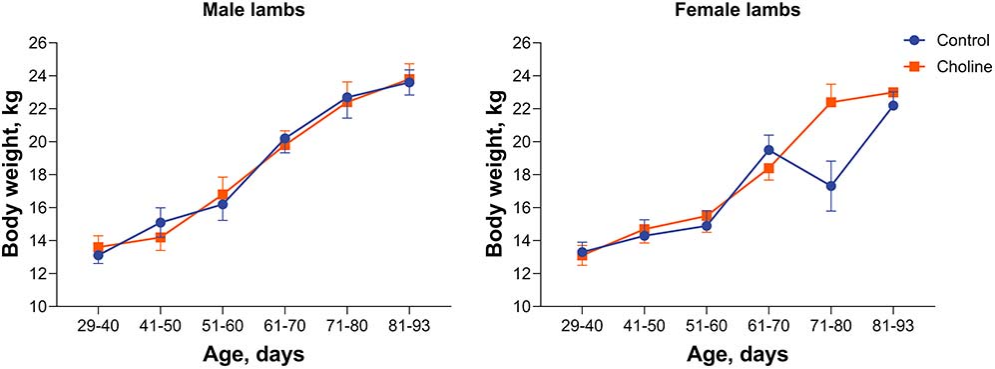
Effect of oral administration of rumen-protected choline during the periconceptional period on body weight of resultant lambs. Data are least-squares mean ± SEM. There was no effect (*P* > 0.10) of rumen-protected choline or the interaction of rumen-protected choline and sex on body weight at any age except for the period from 71 to 80 days in which the interaction was *P* = 0.0142.

There was no effect of treatment (*P* = 0.998) on the percent of lambs that died between birth and weaning (Table 2). Similarly, treatment did not affect body weight from birth until weaning or average daily gain from birth until weaning (Table 2). With one exception, there was no effect of treatment or the treatment by sex interaction for body weight at any of the time points measured (Fig. 2). The exception was for the weights measured from 71 to 80 days of age; lambs were lighter for the control group than for the RPC group for females, but there was no difference between treatments for male lambs.

Male lambs were castrated at an average age of 50 days, and paired testes were collected (Table 2). Paired testes weight from male lambs at castration (average age of 50 days) was not affected by treatment (*P* = 0.982).

### Postnatal development of lambs after weaning as affected by oral administration of rumen-protected choline to females from day −1 to day 7 after anticipated breeding

At an average age of 169 days (range: 152–177 days), data on body weight and carcass characteristics were collected from lambs from replicate 2. Body weight was greater for RPC lambs than for control lambs (*P* = 0.044) (Table 3). The same was true for cross-sectional area (*P* = 0.007) and depth (*P* = 0.019) of the longissimus thoracis muscle as measured by ultrasound. There was no effect of RPC on fat thickness above the muscle (*P* = 0.374) (Table 3)

**Table 3:** Effect of oral administration of rumen-protected choline (RPC) from day −1 to day 7 after anticipated breeding on the average daily gain from birth until weaning and carcass characteristics of lambs at an average age of 169 days. Data are presented as least-square mean ± SEM.

Item	Treatment				*P*-value		
	Control female	RPC female	Control male	RPC male	Treatment	Sex	Treatment × sex
Birth weight, kg*	3.3 ± 0.2	3.2 ± 0.1	3.5 ± 0.2	3.4 ± 0.1	0.593	0.022	0.673
Average daily gain, kg^†^	0.23 ± 0.01	0.25 ± 0.01	0.23 ± 0.01	0.25 ± 0.1	0.785	0.032	0.912
Paired testes weight, g^‡^	-	-	10.0 ± 1.6	10.9 ± 1.5	0.617	-	-
Measurements at 152–177 days of age^§^							
Body weight, kg	28.6 ± 2.2	34.4 ± 1.9	27.7 ± 3.8	35.4 ± 2.4	0.044	0.994	0.606
Longissimus thoracis area, cm^2^	6.8 ± 0.8	9.9 ± 0.6	6.4 ± 1.1	9.7 ± 0.7	0.007	0.717	0.902
Longissimus thoracis depth, cm	2.2 ± 0.1	2.6 ± 0.1	2.1 ± 0.2	2.7 ± 0.1	0.019	.919	0.540
Fat thickness at 12th rib, cm	0.34 ± 0.04	0.41 ± 0.03	0.32 ± 0.06	0.34 ± 0.04	0.374	0.373	0.450

* Data are for lambs of replicates 1–3.

^†^ Data are for all lambs in replicates 1–3 from birth until weaning (average age = 83 days).

^‡^ Measured at castration at an average age of 50 days for ram lambs in replicates 1 and 2 (*n* = 38).

^§^ Only from lambs in replicate 2 (*n* = 55). The average age was 169 days of age.

.

## Discussion

There were two major findings of this experiment. The first was that supplementation with RPC caused a change in sex ratio so that the proportion of lambs that were females was greater than in controls. This difference, which was seen for both singleton and multiple-lamb pregnancies, was not associated with a change in maternal fertility. Such an effect can best be explained by actions of choline to favor fertilization with X-bearing sperm. The second finding was that unlike what occurs in cattle, where oral supplementation of RPC from day −1 to 7 relative to artificial insemination resulted in calves of lighter body weight than calves born from control females (Sagheer *et al.* 2025*b*), oral administration of RPC during the periconceptional period did not affect body weight or average daily gain of lambs before weaning and increased body weight in the post-weaning period.

Given the failure of maternal choline supplementation to alter sex ratio in cattle (Sagheer *et al.* 2025*b*), it can be concluded that supplementation of RPC during the periconceptional period has species-specific effects on the offspring. Either the mechanisms underlying the actions of choline in ewes are distinct from those in cows, lactational status differed between studies, or differences in dosage or metabolism were responsible for divergent results in the two species. One possibility is that the effective dosage of RPC differed between the experiment with cows and sheep. In the experiment with cattle (Sagheer *et al.* 2025*b*), cows were allowed to access the RPC free choice and voluntary consumption was only about half of the planned amount. In contrast, the use of oral boluses in the current experiment ensured that each ewe received the entire planned dose of RPC on each treatment day. It is also possible that species differences represent species-specific variations in the metabolism of RPC or alterations in the rumen microbiome.

While there was no programming effect of choline on lamb growth before weaning, lambs from ewes administered RPC were heavier in the postnatal period at ∼169 days of age and also had increased size of the longissimus thoracis muscle. Calves derived from embryos supplemented with choline exhibited an overrepresentation of genes associated with mTOR signaling in muscle tissue (Estrada-Cortés *et al.* 2021). It is possible that RPC supplementation influenced mTOR signaling patterns in lambs, contributing to enhanced muscle growth.

The action of RPC to skew the sex ratio toward females was not expected because choline supplementation did not affect sex ratio in cattle regardless of whether it was added to embryo culture medium (Estrada-Cortés *et al.* 2021, Haimon *et al.* 2024*a*,*b*) or fed during the periconceptional period (Sagheer *et al.* 2025*b*). Nutritional and other environmental factors have been reported to influence offspring sex ratio (Trivers & Willard 1973, Green *et al.* 2008, Schmidt & Hood 2012, Clayton *et al.* 2015, Purdue-Smithe *et al.* 2021). Conceptually, there are only two possible mechanisms by which RPC could have altered sex ratio – by selectively favoring fertilization by X-bearing gametes or by selectively favoring the survival of female embryos. The latter explanation is unlikely because RPC did not alter fertility or pregnancy loss, and the effect of sex ratio was seen for singleton and multiple-lamb pregnancies. It is possible that RPC affected the hypothalamus, pituitary gland, or ovaries of ewes to modify the timing of ovulation because the time of mating or insemination relative to ovulation has been reported to affect the sex ratio in ewes (Khalifa *et al.* 2013), cows (Pursley *et al.*1998), and golden hamsters (Huck *et al.* 1990). It is also possible that RPC changed the reproductive tract environment in a manner that favored the survival of X-bearing spermatozoa.

An important question is whether actions of orally administered RPC involve increases in choline concentrations in blood or whether other mechanisms are involved. Oral administration of RPC did not result in an increase in plasma concentrations of choline in the present experiment. Previous studies in cattle have reported inconsistent effects of oral RPC supplementation on plasma choline concentrations (De Veth *et al.* 2016, Zenobi *et al.* 2018, France *et al.* 2022, Lima *et al.* 2024, Sagheer *et al.* 2025*b*), which may reflect differences in the degree of ruminal protection and product bioavailability. One study found that the relative bioavailability of RPC was only 13–14% compared with abomasally infused choline chloride (De Veth *et al.* 2016), indicating that RPC may be under- or overprotected. In addition, it is possible that choline is rapidly taken up by tissues following ingestion, which could explain the lack of a clear increase in choline concentrations in plasma after oral administration. The lack of increase in betaine indicates that little of the ingested choline was oxidized to betaine *in vivo*, which in turn means that there was no extra methyl donor available to remethylate homocysteine to methionine. Plasma methionine concentrations were unaffected by RPC, only varying over time (likely due to normal postprandial metabolism).

Consistent with our study, plasma methionine was not affected by RPC feeding in dairy cows (France *et al.* 2022), implying that choline dose did not appreciably spare or supply methionine in the one-carbon metabolic cycle. This result aligns with the concept that choline’s methyl-donor function can spare methionine, but only if enough choline is absorbed to contribute to one-carbon metabolism (Grummer 2013). In ruminants, however, endogenous synthesis via the hepatic phosphatidylethanolamine methyltransferase pathway (which uses methionine-derived methyl groups) typically compensates for extensive ruminal choline losses (Grummer 2013). Thus, a single RPC bolus in these ewes may not have been enough to perturb the tightly regulated one-carbon metabolism and methylation balance.

The TMAO concentrations in blood plasma tended to be greater in RPC-treated ewes. TMAO is a product of gut microbial choline metabolism: intestinal microbes convert choline to trimethylamine, which is then oxidized to TMAO in the liver (Zeisel *et al.* 1983). The observed rise in TMAO concentrations suggests that a portion of the protected choline was still degraded by microbes (possibly due to incomplete rumen protection or hindgut fermentation). This observation agrees with dairy cow studies where greater amounts of RPC led to increased plasma TMAO concentrations (France *et al.* 2022). It also reinforces that degradation of choline to TMAO must be considered when evaluating choline bioavailability from different products.

Concentrations of PAGs in blood were measured at days 30 and 84 of gestation to evaluate possible effects of choline supplementation on placental function. The lack of a difference in PAG concentrations between treatments is similar to what was observed in cattle (Sagheer *et al. 2025b*) but does not necessarily mean that choline did not affect placental function. As expected, concentrations of PAGs increased from day 30 to day 84 and were greater in ewes carrying more than one fetus (Vandaele *et al.* 2005).

In conclusion, oral administration of RPC from day 1 before until day 7 after anticipated breeding did not improve pregnancy outcomes or offspring growth before weaning but skewed the sex ratio toward females and programmed the embryo to increase postnatal growth in the lambs. These effects occurred without a change in plasma concentrations, suggesting that actions of choline are not mediated directly. These results provide further evidence for the environmental modification of sex ratio in mammals and indicate the impact that feeding specific nutrients early in development can have on postnatal phenotype.

## Declaration of interest

The authors declare that no conflict of interest could be perceived as prejudicing the impartiality of the study reported.

## Funding

The research was supported by USDA AFRI grant 2023-67015-40730, the Southeast Milk Checkoff Program, and the L.E. ‘Red’ Larson Endowment. M Sagheer was supported by the Higher Education Commission of Pakistan.

## Author contribution statement

MS and PJH designed the experiment, analyzed the data, and wrote the original draft. All authors helped with data collection and proofreading of the manuscript.
